# Amino acids profiling and transcriptomic data integration demonstrates the dynamic regulation of amino acids synthesis in the leaves of *Cyclocarya paliurus*

**DOI:** 10.7717/peerj.13689

**Published:** 2022-07-05

**Authors:** Zhaokui Du, Weida Lin, Jinxing Zhu, Junmin Li

**Affiliations:** 1 Zhejiang Provincial Key Laboratory of Plant Evolutionary Ecology and Conservation, Taizhou University, Taizhou, Zhejiang, China; 2 Taizhou Vocational College of Science and Technology, Taizhou, Zhejiang, China; 3 Suichang County Bureau of Agriculture and Rural Affairs, Suichang, Zhejiang, China

**Keywords:** *Cyclocarya paliurus*, Amino acids, Developmental stages, Tea, Metabolomics, Transcriptomics

## Abstract

**Background:**

*Cyclocarya paliurus* is a tree well known for its edible and medicinal leaves. Amino acids are essential nutritional components that are present in foods and closely related to the flavor and quality of tea. However, the abundance of amino acids and the regulation of amino acid biosynthesis in the leaves of *C. paliurus* have not been investigated across different developmental stages.

**Methods:**

A combined metabolomic and transcriptomic analysis was employed to investigate the changes in the amino acid profile over several developmental stages (S1, the smallest fully expanded leaf; S3, full leaf enlargement and full leaf thickness; and S2, an intermediate developmental stage between S1 and S3) and the molecular mechanism was elucidated.

**Results:**

The results showed that leaves at the S1 stage had the highest content, while those at the S3 stage had the lowest content of amino acids; fourteen differentially expressed genes were involved in the glycolysis pathway, the tricarboxylic acid cycle and the pentose phosphate pathway, which indicated that the reduced abundance of amino acids in the leaves of *C. paliurus* (mature leaves) may be attributable to reduced gene expression related to carbohydrate metabolism. Four basic leucine zipper transcription factors might play important roles in the regulation of the biosynthesis of amino acids in the leaves of *C. paliurus*.

**Conclusions:**

Leaves at the S1 stage are recommended for high quality tea production because of their high content of amino acids, while leaves at the S2 stage are recommended for generous tea production because of their high levels of sweet flavor amino acids (alanine) and essential amino acids (methionine, phenylalanine, threonine, and tryptophan).

## Introduction

*Cyclocarya paliurus* (Batal) Iljinskaja, a member of Juglandaceae that is commonly known as the “sweet tea tree” due to the sweet flavor of its leaves, is primarily distributed throughout the subtropical areas of China (Zhou et al., 2019a). Pharmacological studies on *C. paliurus* indicated that its leaf extracts have a variety of biological functions, including antioxidant, antimicrobial and antidiabetic activities, for the synergies of abundant phytochemicals, such as flavonoids, triterpenoids, polyphenolics, polysaccharides and other compounds (Liu et al., 2018; Yang et al., 2018; Zhou et al., 2019b). Therefore, a large-scale production of leaves is required for the production of tea and functional food ingredients from *C. paliurus* in China (Fang et al., 2011). Evaluating the characteristics related to tea production may promote the commercial application of *C. paliurus* leaves.

Amino acids are essential nutritional components that are present in foods (Tsochatzis, Papageorgiou & Kalogiannis, 2019) and are closely related with the flavor and quality of tea (Zhang, Liu & Ruan, 2017). Total amino acid content has been utilized as an important criterion for tea quality assurance and contributes to the overall quality of tea in terms of taste and color (Yao et al., 2006). Scharbert, Holzmann & Hofmann (2004) found that aspartate and glutamate taste umami-like; glycine, alanine, glutamine, threonine, and serine taste sweet; and histidine, valine, and tryptophan taste bitter. Yu & Yang (2019) found that glutamine, glutamate, and arginine are the main amino acids in tea. Jabeen et al. (2019) found that amino acids are the main bioactive components of tea beverages and contribute to the flavor of tea. The chlorotic tea varieties *Anji Baicha* and *Huangjingya* contain elevated levels of free amino acids in their leaves, which enhance the drinking quality of their brewed tea (Ma et al., 2012; Zhang, Liu & Ruan, 2017). Except for a study reporting that *C. paliurus* is rich in glutamate, aspartate, leucine, glycine, arginine, tyrosine, alanine, isoleucine, lysine and valine (Xie et al., 2010), scarce detailed information about the amino acid profiling of *C. paliurus* has been reported.

Amino acids are essential molecules incorporated in numerous biochemical reaction pathways in plants (Santiago & Sharkey, 2019). Amino acids are not only the constituents of proteins, but also the precursors of metabolites (Yu & Yang, 2019). For example, arginine, lysine, and methionine are the common precursors of polyamine (Wan & Xia, 2015). Tryptophan and methionine are the precursors for auxin and ethylene, respectively (Wan & Xia, 2015). Glutamate and glutamine contribute to the nitrogen cycle (Yu & Yang, 2019). Amino acids are synthesized in leaves, and their composition varies across different tissues/cell types of plants (Kishor et al., 2015; Rotsch et al., 2017; Santiago & Sharkey, 2019). The concentrations of total free amino acids (including amides) in tea leaves can range from 1% to 5% (Zhang, Liu & Ruan, 2017). The most abundant free amino acids identified in tea plants are theanine, glutamine, glutamate, and arginine (Harbowy et al., 1997). Amino acid content and composition were also observed to vary among different developmental sstages. Nativ et al. (2017) found that the levels of isoleucine, valine, and aspartate increased significantly throughout the developmental stages of *Phelipanche aegyptiaca*, exhibiting the highest levels in mature plants. To the best of our knowledge, the abundance of amino acids and the regulation of amino acid biosynthesis in the leaves of *C. paliurus* have not been investigated across different developmental stages.

The bZIP family is an important transcription factor family in higher plants and is involved in many cell regulation processes (Dröge-Laser et al., 2018). Among all bZIP groups, S1-bZIP group members have been shown to be involved in regulating amino acid metabolism in plants (Ma et al., 2011; Song et al., 2013). For example, AtbZIP1, AtbZIP11 and AtbZIP53 function as important negative regulators of the levels of branched-chain amino acids (BCAAs), including valine, leucine, and isoleucine, and are involved in regulating BCAA degradation (Sun et al., 2020). However, little is known about the role of bZIP family on the amino acids biosynthesis.

Recent advances in various ‘omic’ technologies have enabled quantitative monitoring of the abundance of various biological molecules in a high-throughput manner and thus enabled the measurement of their variations across different biological states on a molecular scale (Zhang, Li & Nie, 2009). Combined analysis of metabolome and transcriptome has been used to explore the biosynthesis of phenolic acid (Lin et al., 2020), flavonoids (Sheng et al., 2021) and polysaccharide (Lin et al., 2021) in the leaves of *C. paliurus*. In this study, amino acids profiling and transcriptomic data were integrated to investigate the changes of amino acids over different developmental stages and attempted to screen out the differentially expressed genes and transcription factors involved in the amino acid synthesis pathway. The results of our study may provide a valuable reference for future efforts attempting to determine the optimal time for the harvest of *C. paliurus* leaves to improve the quality of tea leaves for commercial production.

## Materials and Methods

### Plant materials and sampling

**Figure 1:**
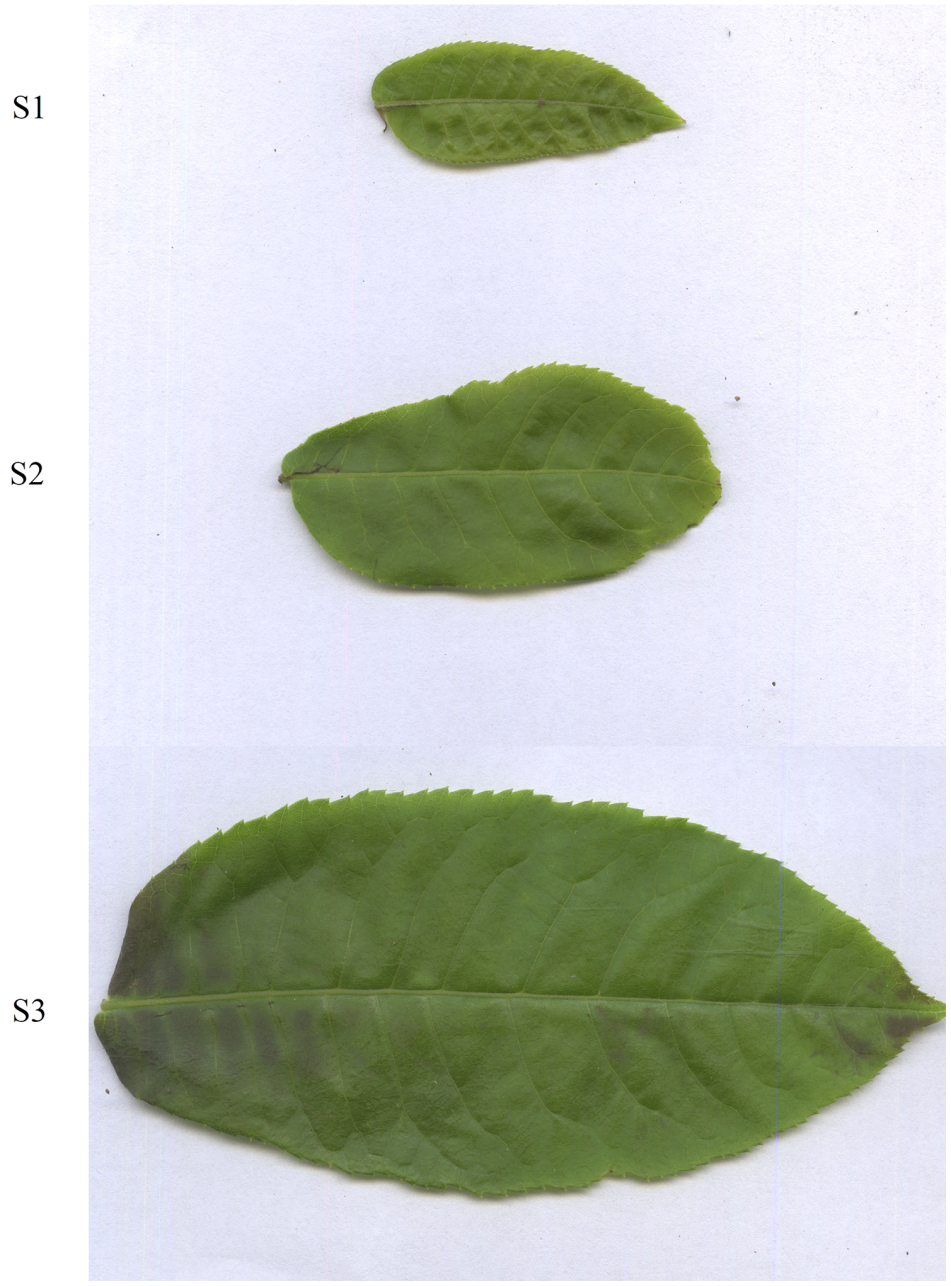
Leaves of *Cyclocarya paliurus* at different developmental stages (S1, S2, S3 stages).

*C. paliurus* leaves at three different developmental stages were collected on 01 May, 2018, in Zhuzhang village, Longquan city, Zhejiang Province, China (E118°48′28″, N28°5′57″). The S1 stage was during the smallest expanded leaf without curly leaf, while the S3 stage was during the largest expanded leaf with full leaf enlargement and full leaf thickness; and S2 stage was an intermediate developmental stage between S1 and S3 stages (Fig. 1). *C. paliurus* leaves were sampled separately on the same tree at the same time, then leaves were divided into three developmental stages according to the leaves’ developmental status and size (Lin et al., 2020). Leaves at S3 stage and S2 stage were significantly larger and longer than those at S2 stage and S1 stage, respectively (Table S1). To obtain sufficient leaves, three samples (three plants) were randomly selected and mixed as one replicate. Three biological replicates (nine plants) were used in this study. The samples were frozen immediately in liquid nitrogen and stored at −80 °C for subsequent metabolite extraction and transcriptome sequencing.

### Metabolite extraction and profiling

Metabolite extraction and identification were conducted by Wuhan Metware Biotechnology Co., Ltd. (Wuhan, China) as previously described in Lin et al. (2020). The freeze-dried leaf was crushed using an MM 400 mixer mill (Retsch, Baltimore, MD, USA) with a zirconia bead for 1.5 min at 30 Hz. One hundred milligrams of *C. paliurus* leaf powder was extracted with 1 mL 70% aqueous methanol solution overnight at 4 °C. After centrifugation at 10,000*g* for 10 min, the extracts were absorbed (CNWBOND Carbon-GCB SPE Cartridge, 250 mg, 3 mL; ANPEL, Shanghai, China) and filtered (SCAA-104, 0.22 μm pore size; ANPEL, Shanghai, China) (Wang et al., 2019). All the samples were mixed and used as the quality control sample (QC) to monitor the precision of the assay.

Next, the sample extracts were analyzed using an LC-ESI-MS/MS system (UPLC, Shim-pack UFLC SHIMADZU CBM30A system; MS, Applied Biosystems 6500 triple quadrupole-linear ion trap mass spectrometer (Q TRAP)) with column (Waters ACQUITY UPLC HSS T3 C18, 1.8 µm, 2.1 mm × 100 mm), equipped with an ESI Turbo Ion-Spray interface, operating in both positive and negative ion mode and controlled using Analyst 1.6 software (AB Sciex, Redwood, CA, USA). Solvent A was water with 0.04% acetic acid and solvent B was acetonitrile with 0.04% acetic acid. The mobile phase was consisted of solvents A and B. A gradient program was executed in the following steps at 0, 11, 12.1 min: 95:5 (v/v), 5:95 (v/v), 95:5 (v/v) at 12.1 min. The flow rate was 0.4 mL/min, the temperature was 40 °C, and the injection volume was 2 μL. The effluent was alternatively connected to an ESI Q-TRAP (AB Sciex QTRAP 6500 System, AB SCIEX Pet. Ltd, Framingham, MA, USA) (Zhang, Lu & Liu, 2022). The parameters of ESI source operation were set as follows: ion spray voltage (IS, 5,500 V), ion source (turbo spray), source temperature (500 °C), ion source gas I (GSI, 55 psi), gas II (GSII, 60 psi), and curtain gas (CUR, 25 psi), and the collision gas (high). Instrument tuning and mass calibration were performed with 10 μmol/L polypropylene glycol solutions in QQQ and 100 μmol/L in LIT modes. Multiple reaction monitoring (MRM) experiments with collision gas (nitrogen) at 5 psi were performed to obtain QQQ scans (Li et al., 2017). The declustering potential (DP) and collision energy (CE) for individual MRM transitions were measured, then DP and CE were further optimized. For each period, MRM transitions were monitored on the metabolites in elution (Dong et al., 2019). Metabolites were annotated according to the information from the public metabolite database and the MetWare database (MetWare Biotechnology Co., Ltd., Wuhan, China). All mass spectrum peaks were subjected to area integration, which represents the relative content of the corresponding metabolites (Zhang, Lu & Liu, 2022).

Fisher least significant difference (LSD) *post hoc* test was used to compare the amino acids content at different developmental stages. Variable importance in projection (VIP) ≥ 1 and log_2_ |fold change| ≥ 1 were set as the criteria to screen the significant differentially accumulated amino acids. Each sample was performed in triplicate. Principal component analysis (PCA) of free amino acids and quality control samples was conducted by Origin (version 2018).

### Transcriptomic analysis

Data were collected as previously described in Lin et al. (2020). Specifically, total RNA was extracted from frozen leaf samples using the Total RNA Extractor (TRIzol) kit (B511311; Sangon, Shanghai, China). The quality and concentration of RNA were determined using a NanoDrop 2000 (Thermo Fisher, Waltham, MA, USA). Two micrograms of the RNA was used for library construction by the VAHTSTM mRNA-seq v2 Library Prep Kit (Illumina, San Diego, CA, USA). First-strand cDNA was synthesized by M-MuLV reverse transcriptase using random hexamer primers, while second-strand cDNA was synthesized by DNA polymerase I and RNase H. The left overhangs were turned into blunt ends by exonuclease/polymerase. AMPure XP system was used to purify library fragments (Beckman Coulter Company, Beverly, MA, USA). PCRs were performed with Phusion High-Fidelity DNA polymerase (Thermo Fisher Scientific Inc., Waltham, MA, USA), universal PCR primers, and Index (X) Primer. PCR products were purified (AMPure XP system), and the quality of the library was determined using a Bioanalyzer 2100 system (Agilent Technologies Inc., Santa Clara, CA, USA). Paired-end sequencing of these libraries was performed on HiSeq X Ten sequencers (Illumina, San Diego, CA, USA) by Novagen Co., Ltd. (Beijing, China). Raw reads of high-throughput sequencing were obtained in the FASTQ file format. *De novo* assembly of the clean reads were conducted by Trinity (version 2.0.6) (parameter: min_kmer_cov 2) (Trinity Technologies, Irvine, CA, USA). To decrease redundancy, we clustered the transcripts with a minimum length of 200 bp. The longest sequence was preserved and designated a unigene for each cluster (Lin et al., 2020). Read alignment statistics and sample quality features were calculated with SAMtools and RSeQC (version 2.6.1) (Lin et al., 2020).

BLAST program was used to annotate the functions of unigenes against protein databases, including NCBI Nr (NCBI non-redundant protein sequences), Swiss-Prot protein, TrEMBL, ConserGene Ontology (GO), CDD (Conserved Domain Database), Pfam, and KOG (eukaryotic Orthologous Groups) database (e-value < 1 ×1 0^−5^). GO (Gene Ontology Database) function annotation information was obtained according to the Uniprot annotation results based on the annotation results of Swiss-+Prot and TrEMBL database. KEGG annotation was conducted by KAAS (KEGG automatic annotation server) (version 2.1). All the assembled unigenes were classified into KEGG pathways using BLASTX against the KEGG database (Zhang et al., 2018). Transcripts per million (TPM) was used to eliminate the influence of gene lengths and sequencing discrepancies (Jiang et al., 2019). DESeq2 (version 1.12.4) was conducted to determine the differentially expressed genes (DEGs) between the two samples at a *q*-value < 0.001 and log_2_ |fold change| ≥ 1 (Lin et al., 2020). Each sample was performed in triplicate. PCA of DEGs was conducted by Origin (version 2018).

### Reverse transcription-quantitative PCR (RT-qPCR)

Sixteen candidate genes involved in glycolysis, the tricarboxylic acid cycle (TCA) and amino acid anabolism were chosen and RT-qPCR was conducted to verify the gene expression. The specific primers were designed using Primer Premier 5.0 (Table S2). Total RNA was reverse-transcribed using HiScript II reverse transcriptase according to the manufacturer’s instructions (Vazyme, Nanjing, China). Real-time PCR experiments were performed using a CFX Connect Real-Time PCR system (Bio-Rad Laboratories Inc., Hercules, CA, USA). β-Actin was used as the internal standard gene (Zhao et al., 2018; Lin et al., 2021). The relative expression of genes was calculated by the 2^−∆∆Ct^ method (Salvatierra et al., 2010). All RT-qPCRs were performed with three biological replicates. All statistical analyses were conducted by SPSS 17.0 software (SPSS Inc. Chicago, IL USA). The data are displayed as the means ± standard deviations (SD).

### Integrated analysis of transcriptomic and amino acids data

The amino acids and transcriptomic data were natural logarithm transformed, and the Pearson correlation coefficient (PCC) between amino acids and transcriptomic data was calculated by the cor function in R (version 3.5.1). |PCC| > 0.8 and *p* < 0.05 were set as the cutoff criteria to assess the association between amino acids and DEGs in *C. paliurus* leaves over three different developmental stages. Finally, the network was visualized through CytoScape (version 3.6.1) (Cho et al., 2016).

### Prediction of key transcription factors and phylogenetic analysis

The key transcription factors (TFs) involved in amino acid biosynthesis were predicted by PlantTFDB (http://planttfdb.gao-lab.org/) (Jin et al., 2017). The expression data were natural logarithm transformed, and the correlation between differentially expressed TFs and other DEGs was calculated by the Corrplot package in R (version 3.5.1) with PCC > 0.8, and *p*-value < 0.05 were considered to be significant. A visual network graph was generated with Cytoscape software (version 3.6.1).

The protein sequences of *C. paliurus* TFs were translated from coding sequences in the transcriptome. The bZIP family protein sequences of *Arabidopsis thaliana* and rice (*Oryza sativa japonica*) were downloaded from NCBI. Phylogenetic and molecular evolutionary analyses were conducted using MEGA version 5.05 with pairwise distance and the maximum-likelihood algorithm. The reliability of each tree was established by performing 1,000 bootstrap sampling steps.

## Results

### Free amino acid profiling in leaves of *C. paliurus*

Metabolite analysis annotated 694 metabolites in S1, S2 and S3 stages, each of which was analyzed using three biological replicates (Table S3). Of the 694 identified metabolites, organic acids (69, 9.94%), nucleotides and their derivatives (59, 8.50%), flavones (57, 8.21%), amino acid derivatives (53, 7.64%) and flavonols (44, 6.34%) accounted for a large proportion (Fig. S1).

**Figure 2:**
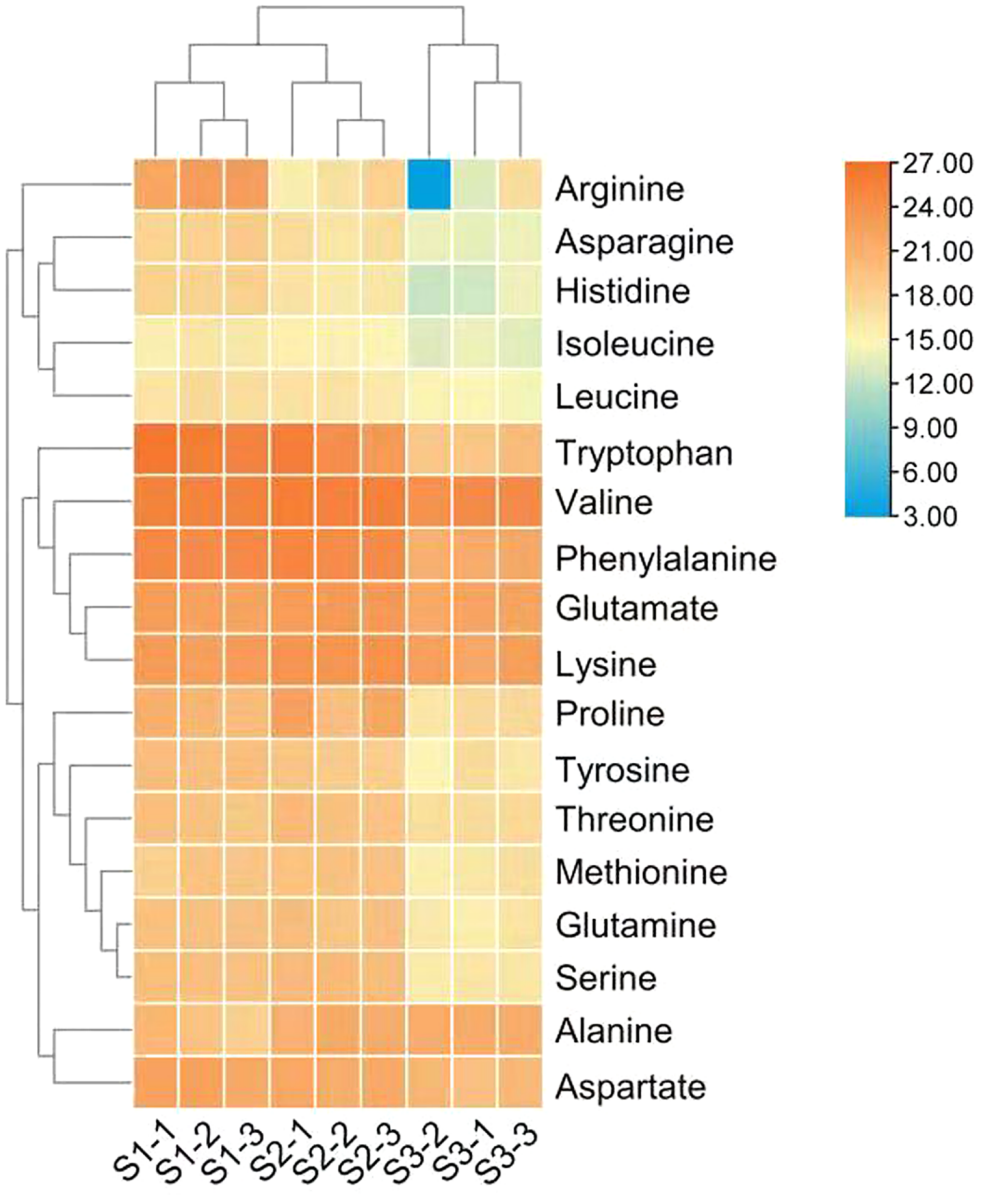
Heat map of free amino acid contents in the leaves of *Cyclocarya paliurus* at different developmental stages. The log2 transformed values of the relative abundance of amino acids are indicated from blue to red (low to high) across the S1, S2 and S3 developmental stages.

The differences in the relative abundance of free amino acid in the leaves of *C. paliurus* at different developmental stages are shown in Table 1 and Fig. 2 and Table S4. A total of 18 amino acids were detected. Regarding total amino acids, the relative abundance of amino acids in the leaves at the S1 stage and S2 stage was significantly higher than that at the S3 stage (Table 1).When the threshold for a significant difference was set at variable importance in projection (VIP) ≥ 1.0 and log_2_ |fold change| ≥ 1, from the S1 stage to the S2 stage, valine and tryptophan in the leaves of *C. paliurus* at the S1 stage were 3.6-fold and 43-fold lower, while alanine was 3.4-fold higher, than at the S2 stage; from the S2 stage to the S3 stage, the contents of eleven amino acids decreased significantly (Table 1). PCA results showed that there were significant differences in the amino acid content among the leaves at different developmental stages (Fig. S2).

**Table 1:** Counts per second of free amino acids in the leaves of *Cyclocarya paliurus* at the S1, S2 and S3 developmental stages.

Amino acids	Count per second												S1 *vs* S2		S2 *vs* S3	
	S1-1	S1-2	S1-3	S2-1	S2-2	S2-3	S3-1	S3-2	S3-3	S1	S2	S3	Fold change	VIP	Fold change	VIP
Alanine	156	69.6	33.4	213	355	308	331	308	322	86.3 ± 63a	292 ± 72b	320 ± 12b	3.38	1.13	/	/
Arginine	455	745	736	5.49	11.8	28.6	1.05	0	14.8	645 ± 165a	15.3 ± 12b	5.28 ± 8c	0.02	2.07	0.35	1.25
Asparagine	22.9	31	43.6	16.2	9.76	14	1.55	1.76	1.82	32.5 ± 10a	13.3 ± 3b	1.71 ± 0.1c	/	/	0.13	1.2
Aspartic acid	589	626	369	429	298	357	84.4	164	152	528 ± 139a	361 ± 66a	133 ± 43b	/	/	/	/
Glutamic acid	819	618	538	796	1010	1010	528	370	550	658 ± 145a	939 ± 124b	483 ± 98a	/	/	/	/
Glutamine	84.8	94.7	91.5	110	65.9	98.1	4.97	7.57	9.83	90.3 ± 5a	91.3 ± 23a	7.46 ± 2b	/	/	0.08	1.33
Histidine	32.3	28	34.6	10.9	6.78	9.35	0.76	0.62	1.98	31.6 ± 3a	9.01 ± 2b	1.12 ± 0.7c	0.28	1.17	0.12	1.22
Isoleucine	4.84	9.65	7.67	4.69	4.56	3.42	1.92	1.08	1.24	7.39 ± 2a	4.22 ± 0.7b	1.41 ± 0.4c	/	/	/	/
Leucine	9.31	19.1	15.2	11.2	11.8	7.09	3	3.99	2.55	14.5 ± 5a	10.0 ± 3a	3.18 ± 0.7b	/	/	/	/
Lysine	933	690	923	1380	1330	1660	409	648	685	849 ± 138a	1,460 ± 178b	581 ± 150a	/	/	/	/
Methionine	33.2	68.1	63.9	67.6	83.9	85.4	8.49	4.78	14.5	55.1 ± 19a	79.0 ± 10a	9.26 ± 5b	/	/	0.12	1.24
Phenylalanine	2730	2360	2630	3370	2340	2280	336	234	390	2,573 ± 191a	2,663 ± 613a	320 ± 79b	/	/	0.12	1.22
Proline	252	165	115	618	101	394	18.8	8.05	18.4	177 ± 69a	371 ± 259b	15.1 ± 6a	/	/	0.04	1.42
Serine	107	96	82.6	120	132	115	9.44	6.12	8.31	95.2 ± 12a	122 ± 9b	7.96 ± 2c	/	/	0.07	1.4
Threonine	90	74.3	57	147	87.6	70.9	16.7	14.6	19.7	73.8 ± 17a	102 ± 40a	17.0 ± 3b	/	/	0.17	1.1
Tryptophan	9,800	5,600	4,030	5,960	2,160	944	57.9	56.8	130	6,480 ± 2,983a	3,020 ± 2,617b	81.6 ± 42c	/	/	0.03	1.52
Tyrosine	113	97.3	107	64.5	43.8	38.3	16.2	3.49	8.42	106 ± 8a	48.9 ± 14b	9.37 ± 6c	/	/	0.19	1.07
Valine	4,500	4,370	4,160	5,730	4,900	4,910	2,470	1,810	2,560	4,343 ± 172a	5,180 ± 476b	2,280 ± 410c	/	/	/	/
Total	20,731	15,762	14,037	19,054	12,952	12,333	4,299	3,644	4,891	16843 ± 3,475a	14,779 ± 3,715a	4,278 ± 624b	/	/	/	/

**Note:**

VIP, variable importance in projection. The results of Fisher leastsignificant difference (LSD) *post hoc* test were shown by small letters. The same letters indicate there is no significant difference between two different developmental stages.

All the counts per second in the table should be multiplied by 10^4^.

### DEGs involved in the amino acid biosynthesis pathway

Transcriptome sequencing of *C. paliurus* in the S1, S2 and S3 stages generated average numbers of 52,453,341, 48,977,149, and 46,906,739 raw reads, respectively. In all the libraries, 93.59% of the sequences had quality scores greater than Q30. After filtering out the low-quality reads, 49,806,827, 46,320,477, and 42,536,039 clean reads were obtained from the S1, S2 and S3 stages with GC contents of 49.28%, 51.91%, and 52.63%, respectively. A total of 296,593 unigenes were obtained with an average length of 430.11 bp and N50 of 478 bp (Table S5).

**Figure 3:**
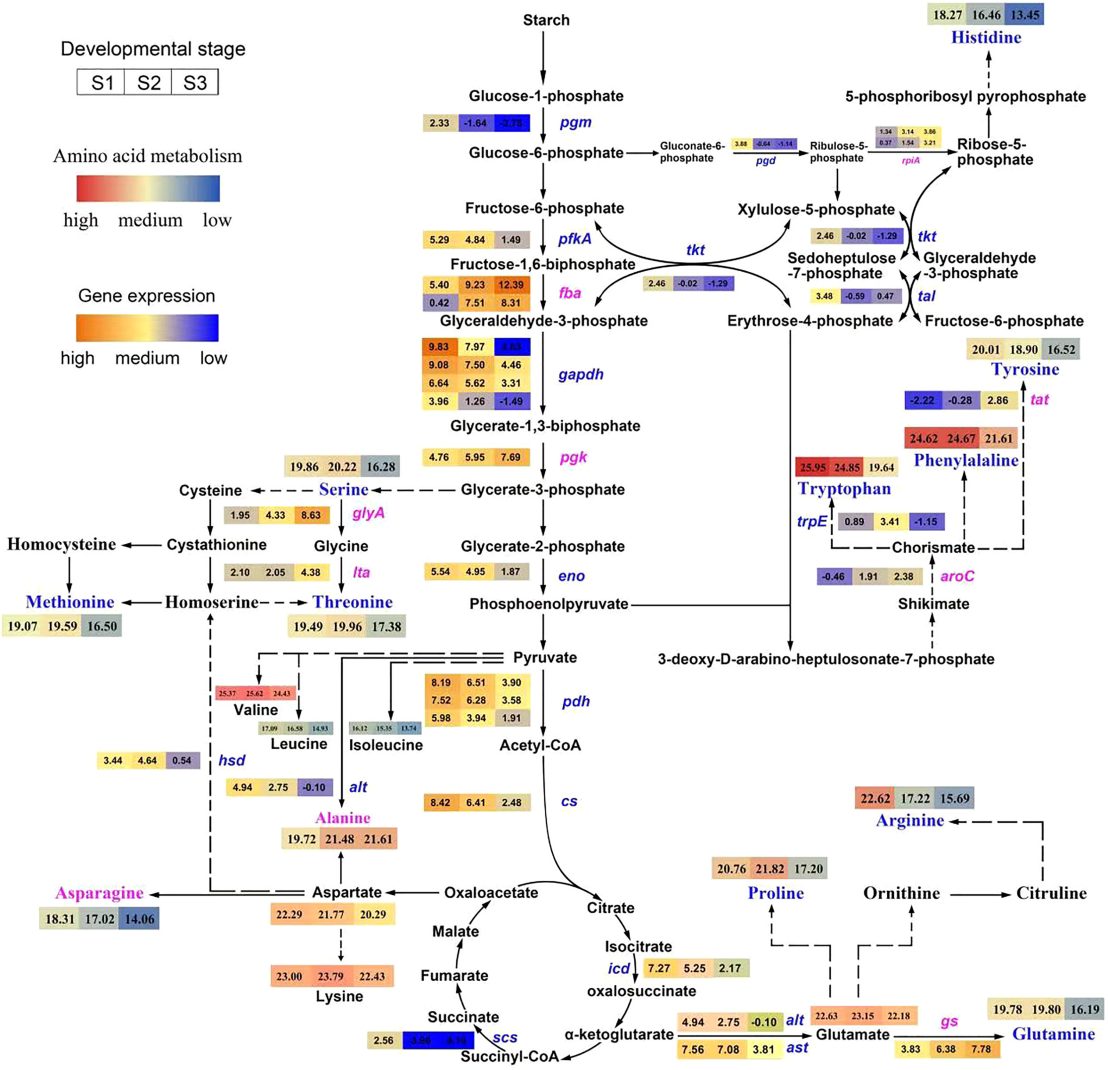
Pathway of amino acid biosynthesis in *Cyclocarya* *paliurus*. The purple coloration of enzyme names indicates a significant upregulation from the S1 to S3 stage in the synthesis of amino acids, and blue indicates a significant downregulation. The log_2_ transformed values of DEGs based on transcripts per kilobase (TPM) value of unigenes of the enzymes are also indicated as changing from blue to red (low to high) across the S1, S2, and S3 developmental stages. The log_2_ transformed values of amino acids are indicated as changing from blue to red (low to high) across the S1, S2, and S3 developmental stages.

A total of 30 DEGs were involved in the amino acid biosynthesis pathway (KEGG map01230, https://www.genome.jp/entry/map01230, Fig. S3 and Fig. 3, Table S4). PCA results showed that there were significant differences in the DEGs among the leaves at different developmental stages (Fig. S4).

Among ten DEGs related to the glycolysis pathway, *pgm*, *pfkA*, *gapdh*, and *eno*, which encode phosphoglucomutase, 6-phosphofructokinase 1, glyceraldehyde 3-phosphate dehydrogenase and enolase, respectively, were downregulated, while *fba* and *pgk*, which encode fructose-bisphosphate aldolase and phosphoglycerate kinase, respectively, were upregulated.

Among six DEGs related to the TCA cycle, the *pgh*, *cs*, *icd* and *scs* genes, encoding pyruvate dehydrogenase, citrate synthase, isocitrate dehydrogenase, and succinyl-CoA synthetase, respectively, were downregulated. Among five DEGs related to the pentose phosphate pathway, the *rpiA* gene, encoding ribose 5-phosphate isomerase A, was upregulated; however, the other three genes, *pgd*, *tkt*, and *tal*, encoding 6-phosphogluconate dehydrogenase, transketolase, and transaldolase, respectively, were downregulated.

Among nine DEGs relate to the amino acids metabolism pathway, the *glyA*, *ltaE*, *gs*, *aroC*, and *tat* gene, encoding glycine hydroxymethyltransferase, threonine aldolase, glutamine synthetase, chorismate synthase, and tyrosine aminotransferase, were upregulated; however, the other four gene, *hsd, alt*, *ast*, *trpE*, encoding homoserine dehydrogenase 1, alanine transaminase, aspartate aminotransferase, and anthranilate synthase component I, were downregulated.

### Validation of the gene expression profiles by RT-qPCR

To validate the transcription profile obtained by RNA-seq data, 16 genes from starch and amino acid metabolism were selected to design specific primers (Table S2) for RT-qPCR analysis. The results indicated that most of the selected genes (except for the *PKAA*, *ENO* and *LTAE* genes) had similar expression patterns to those identified through RNA-seq analysis (Fig. S5). The overall correlation coefficient (R^2^ = 0.64) between RNA-seq and RT-qPCR data was obtained by linear regression analysis (*p* < 0.0001) and exhibited a good correlation between these two datasets (Fig. S6, Table S8), indicating that the nine transcriptomic datasets were credible.

### Integrated analysis between DEGs and amino acids

**Figure 4:**
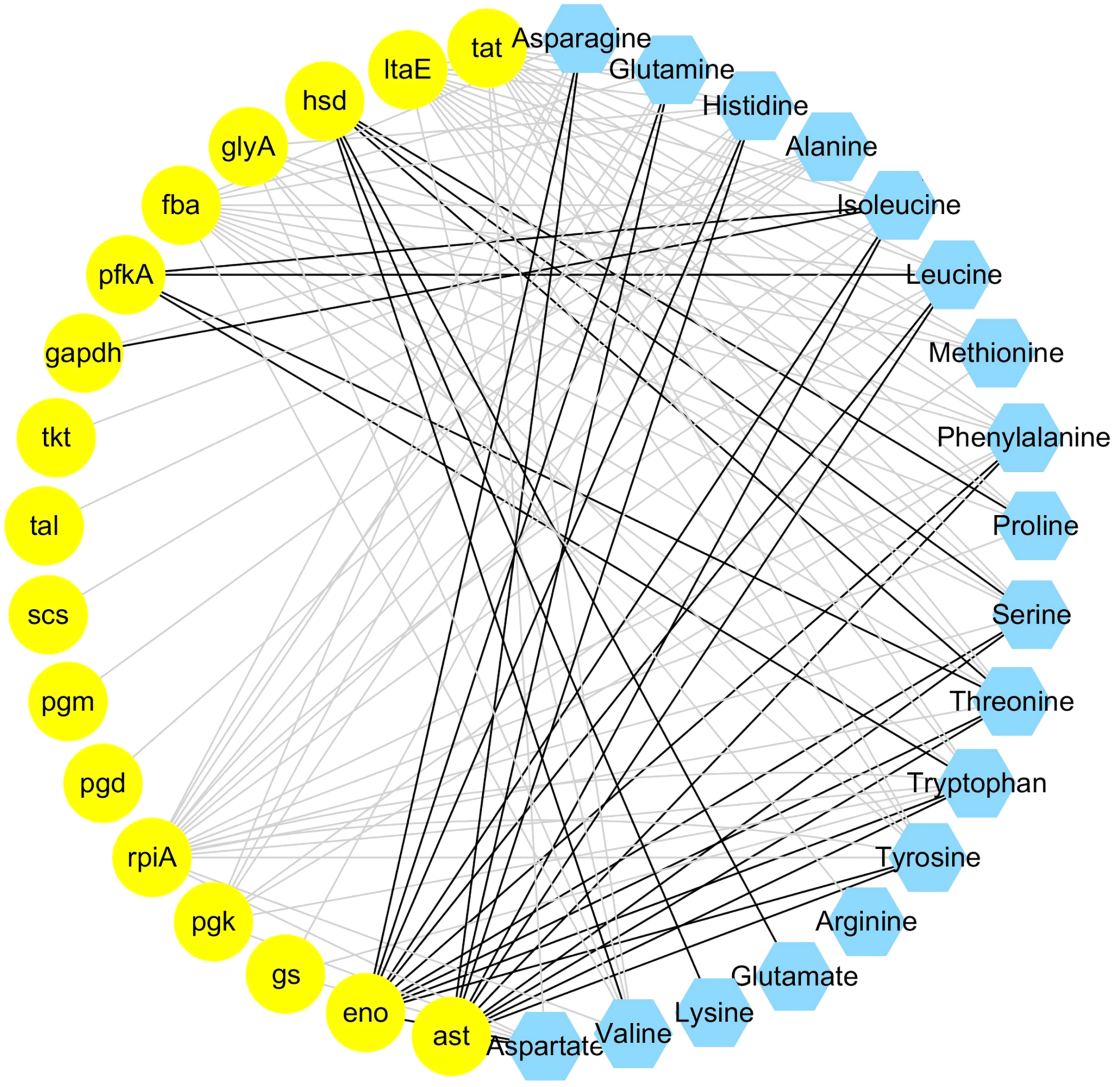
Coexpression analysis of DEGs and amino acids in the leaves of *Cyclocarya paliurus* at different developmental stages. The yellow circles represent DEGs, and the blue hexagon represents amino acids. Black and grey edges represent positive and negative correlations.

The integrated analysis of the log_2_-transformed amino acids and transcriptomic data showed that 34 DEGs correlated significantly with 18 amino acids, resulting a total of 110 related pairs (34 positive and 76 negative) between the DEGs and amino acids (Fig. 4, Table S6). Seventeen genes were determined to be involved in involved in the amino acid biosynthesis pathway. Among them, six genes (*pgm*, *pfkA*, *fba*, *gapdh*, *pgk*, *eno*) are related with the glycolysis pathway, one gene (*scs*) is related with TCA cycle, four genes (*pgd*, *rpiA*, *tkt*, *tal*) are related with pentose phosphate pathway and six genes (*tat*, *ltaE*, *hsd*, *glyA*, *gs*), and *ast* are related with amino acids metabolism (Fig. 4).

### Screening of transcription factors related to amino acid biosynthesis

**Figure 5:**
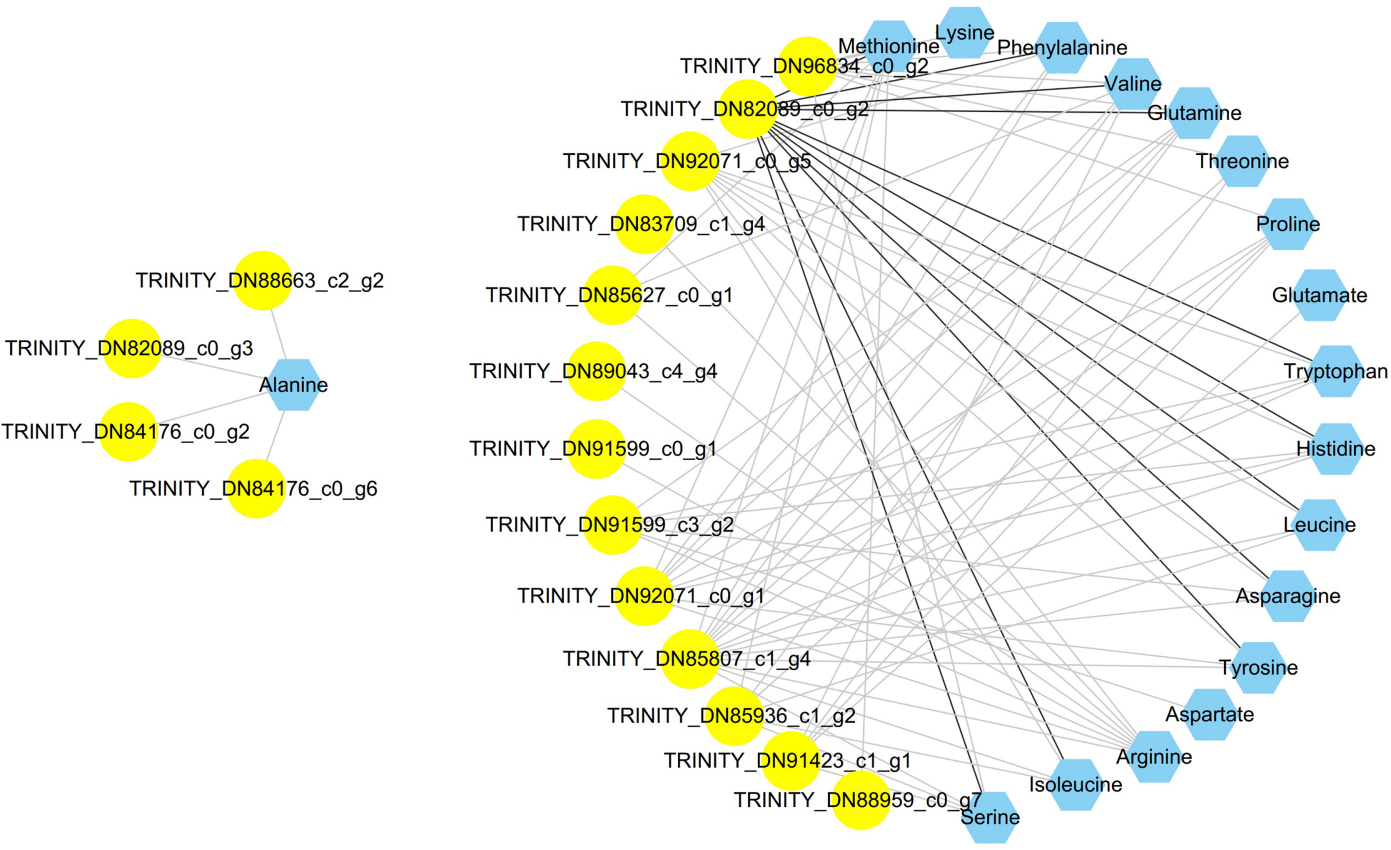
Coexpression analysis of bZIP genes and amino acids in the leaves of *Cyclocarya paliurus* over different developmental stages. Genes are shown in yellow circles, amino acids are shown in blue hexagons, black lines represent positive correlations, and grey lines represent negative correlations.

To clearly determine the unknown putative transcription factors controlling the amino acid biosynthesis of *C. paliurus*, coexpression analysis was employed between amino acids and differentially expressed transcription factor bZIP genes. A total of 60 differentially expressed bZIP genes were identified *via* comparison with PlantTFDB (Table S9). A total of 78 related pairs were identified. The visualized network showed that a total of 35 nodes were connected by 78 edges (Fig. 5). Only TRINITY_DN82089_c0_g2 had a positive correlation with 11 amino acids; however, the other bZIP genes were negative for amino acids.

### Phylogenetic analysis of bZIP family members in *C. paliurus*

**Figure 6:**
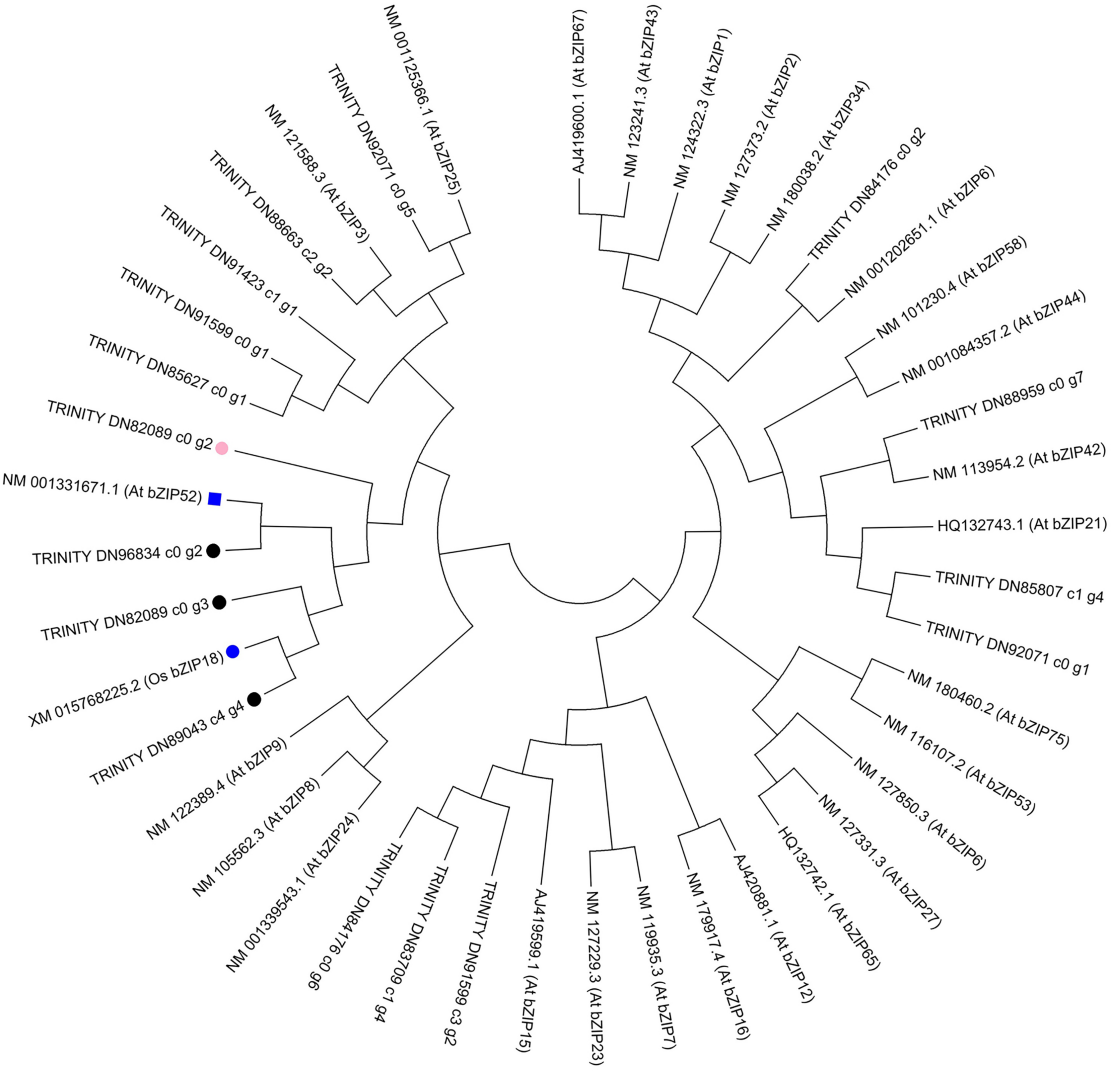
Phylogenetic analysis of DEGs associated with bZIPs in *Arabidopsis thaliana* (At) and *Oryza sativa* (Os). The pink circle indicates a transcription factor that was positively correlated with the biosynthesis of amino acids in *Cyclocarya paliurus*. The black circles indicate transcription factors that were negatively correlated with the biosynthesis of amino acids in *Cyclocarya paliurus*. At *bZIP52* and Os *bZIP18* are indicated by the blue box and blue circle, respectively.

To further investigate the evolution of the *C. paliurus* bZIPs, we constructed phylogenetic trees either from the full-length *C. paliurus* bZIPs or from the bZIP domains alone. As shown in Fig. 6, TRINITY_DN89043_c4_g4 clustered with *OsbZIP18* and subsequently clustered with TRINITY_DN82089_c0_g3. TRINITY_DN96834_c0_g2 clustered with *AtbZIP52*. Both of these bZIPs clustered together and then clustered with TRINITY_DN82089_c0_g2.

## Discussion

Amino acids play important roles in the flavor and quality of tea, and each free amino acid has its own taste, such as sweet, salty, sour, bitter, and umami; therefore, free amino acids are considered to be the primary factors affecting the mellow taste of green tea (Horanni & Engelhardt, 2013; Yu & Yang, 2019), white (Horanni & Engelhardt, 2013; Chen et al., 2021), black, oolong, and pu-erh teas (Horanni & Engelhardt, 2013). The profiling of free amino acids is also considered to be an important criterion for tea or fruit quality assurance (Yao et al., 2006; Xu et al., 2020). In this study, we found that a total of 18 amino acids were detected in the leaves of *C. paliurus*, and the amino acids contents in the youngest leaves at the S1 stage and S2 stage were significantly higher than those in the S3 stage. Usually, glutamine, glutamic acid and arginine are the most abundant free amino acids in tea plants (Harbowy et al., 1997); however, the contents of amino acids in tea plants are highly influenced by plant variety, nutrition and the local environment (Zhang, Liu & Ruan, 2017). In this study, according to the relative abundance of amino acids, we found that tryptophan, valine, and phenylalanine were the top three free amino acids in the S1 stage; while valine, tryptophan, and lysine were the top three in the S2 stage; and valine, lysine and glutamic acid were the top threein the S3 stage. The Fisher LSD *post hoc* test results showed that arginine and histidine in S2 stage were significantly lower, while alanine, glutamic acid, lysine, proline, serine, and valine were significantly higher than those in S1 stage; all amino acids in S3 stage, with the exception of alanine, were significantly lower than those in S2 stage (Table 1). To the best of our knowledge, this report is the first to describe the dynamic changes in amino acids over different developmental stages of *C. paliurus* leaves. It was reported that alanine is the most sweet amino acid in the L-form (Solms, 1969), and glutamic acid is responsible for the umami taste of green tea (Kaneko et al., 2006; Horanni & Engelhardt, 2013). Essential amino acids, including leucine, isoleucine, histidine, valine, tryptophan, phenylalanine, lysine, methionine, and threonine, can only be derived from dietary protein (Stoll et al., 1998). In this study, alanine at S2 stage and S3 stage were 3.38 fold and 3.71 fold higher than that at S1 stage, respectively. Therefore, leaves at the S1 and S2 stages were recommended for tea production because of their high content of amino acids, especially leaves at the S2 stage were recommended for their high levels of sweet flavor amino acids (alanine) and essential amino acids, including valine, methionine, phenylalanine, lysine, threonine, and tryptophan. Leaves at the S3 stage are recommended for the industrial production of polysaccharides (Lin et al., 2021), phenolic acids (Lin et al., 2020) and flavonoids (Sheng et al., 2021) from the leaves of *C. paliurus*.

The increase in the content and composition of amino acids in plants might be due to increased protein degradation (Zhang, Liu & Ruan, 2017; Chen et al., 2021; Duan et al., 2021), decreased glycolysis, increased biosynthesis of amino acids and the activation of the matabolism of other nitrogen-containing compounds, including chlorophyll, purines, nucleotides and alkaloids (Zhang, Liu & Ruan, 2017). In this study, we found that most DEGs were involved in the glycolysis pathway, the TCA cycle and the pentose phosphate pathway (PPP), which indicated that the reduced abundance of amino acids in the mature leaves of *C. paliurus* may be due to reduced gene expression related to carbohydrate metabolism. A similar mechanism was reported by Wang, Ma & Cheng (2010) for chlorotic leaves of *Malus domestica*, which had lower concentrations of many individual free amino acids compared with normal leaves due to significantly decreased concentrations of most intermediates in glycolysis and the TCA cycle.

The glycolysis-TCA cycle is a major energy source and provides a carbon skeleton for the synthesis of fundamental metabolites, plant growth and development (Fernie, Carrari & Sweetlove, 2004; Wang et al., 2018; Saavedra et al., 2019; Park et al., 2017). Glycolysis is an oxygen-independent pathway that generates adenosine triphosphate (ATP), nicotinamide adenine dinucleotide (NADH), and pyruvate and produces building blocks for anabolism (Plaxton, 1996). Aerobic organisms use the TCA cycle to release stored energy through the oxidation of acetyl-CoA (Sweetlove et al., 2010). In addition, the cycle provides precursors of certain amino acids, as well as the reducing agent NADH, which are used in numerous other reactions. Cramer et al. (2013) demonstrated that enhancing the expression of glycolysis-related enzymes could increase the synthesis of certain amino acids, such as glutamate, threonine, glycine, and cysteine. Glaubitz et al. (2015) reported that the levels of nine amino acids (three from the oxaloacetate/aspartate family and two each from the α-ketoglutarate, 3-phosphoglycerate and pyruvate families) were significantly higher in *Oryza sativa* combined with parallel increases in the TCA cycle intermediate malic acid. Sun et al. (2016) showed that wild ginseng had enhanced amino acid contents and higher expression of enzymes related to glycolysis and TCA compared with cultivated ginseng. Furthermore, the biosynthesis of amino acids, particularly aromatic amino acids, such as phenylalanine, tyrosine, and tryptophan, is associated with chorismate, which condenses phosphoenolpyruvate (PEP) from glycolysis and erythrose 4-phosphate (E4P) from PPP (Bromke, 2013). In this study, both the decreased abundance of intermediates in carbohydrate metabolism and the reduced expression of several key enzymes gene (*pgm*, *pfkA*, *gapdh*, *eno*, *pgh*, *cs*, *icd*, *scs*, *pgd*, *tkt*, and *tal*) clearly indicated that both glycolysis and the TCA cycle were downregulated in *C. paliurus* mature leaves compared with young leaves. Zhao et al. (2016) also observed that TCA cycle intermediates were closely correlated with amino acid pools in Yunnan tobacco, and proline and asparagine were significantly associated with TCA cycle intermediates in Guizhou tobacco.

Integrated analysis between DEGs and amino acids also indicated that the reduced abundance of amino acids in the leaves of *C. paliurus* may be attributable to reduced gene expression related to carbohydrate metabolism. For example, the content of alanine was negatively correlated with the expression of genes related with glycolysis pathway (*pgm*, *gapdh*), TCA cycle (*scs*), and pentose phosphate pathway (*pgd*, *tkt*, *tal*). Integrated analysis between DEGs and amino acids also showed that two genes (*hsd*, *ast*) are positively correlated with amino acid content. Aspartate aminotransferase, encoded by *ast* gene, catalyzes the specific biosynthesis of aspartate (Jansen et al., 2020). Homoserine dehydrogenase 1, encoded by *hsd* gene, plays important role in threonine biosynthesis (Ferreira, Meinhardt & Azevedo, 2006). The downregulated expression of *hsd* and ast genes indicated that the reduction of amino acids also be regulated by key enzymes related with amino acids biosynthesis.

Phylogenetic analysis in our research showed that TRINITY_DN89043_c4_g4 clustered with *OsbZIP18* and subsequently clustered with TRINITY_DN82089_c0_g3. TRINITY_DN96834_c0_g2 clustered with *AtbZIP52*. Both of the bZIPs clustered together and later clustered with TRINITY_DN82089_c0_g2. Recently, Sun et al. (2020) reported that the transcription factor OsbZIP18 in rice can positively regulate the biosynthesis of BCAA by directly modulating the key genes *OsBCAT1* and *OsBCAT2*. *bZIP52* is a close homolog of *bZIP18* and is an interacting partner of *bZIP18* (Wiese et al., 2021). From S1 stage to S2 stage, then S3 stage, the expression level of TRINITY_DN82089_c0_g2 gene decreased from 3.21 to 2.92, then 0.69; that of TRINITY_DN89043_c4_g4 increased from 3.54 to 5.67, then 6.75; that of TRINITY_DN82089_c0_g3 increased from 32.59 to 11.45, then 7.27; that of TRINITY_DN96834_c0_g2 decreased from 7.14 to 2.86, then increased to 13.52. Coexpression analysis showed that the TRINITY_DN82089_c0_g2 gene had a positive correlation with the amino acid content, while the other three bZIP TF were negatively correlated with the amino acid content. Therefore, we can predict that the interactions between bZIP TFs TRINITY_DN82089_c0_g2, TRINITY_DN89043_c4_g4, TRINITY_DN82089_c0_g3, and TRINITY_DN96843_c0_g2 might play important roles in the regulation of the biosynthesis of amino acids in the leaves of *C. paliurus* during the developmental stages. These results may help to elucidate amino acid biosynthesis during the development of leaves of *C. paliurus*. Further studies based on gene cloning are warranted to determine the precise roles played by bZIP TFs in the regulation of the biosynthesis of amino acids.

## Conclusions

Leaves at the S1 stage had the highest content of amino acids, while those at the S3 stage had the lowest content of amino acids. Leaves at the S1 stage are recommended for high quality tea production because of their high content of amino acids, while leaves at the S2 stage are recommended for generous tea production because of their high levels of sweet flavor amino acids (alanine) and essential amino acids (methionine, phenylalanine, threonine, and tryptophan). Fourteen DEGs were involved in the glycolysis pathway, the tricarboxylic acid cycle (TCA) and the pentose phosphate pathway (PPP), which indicated that the reduced abundance of amino acids in the mature leaves of *C. paliurus* may be attributable to reduced gene expression related to carbohydrate metabolism. Among these genes, *pfkA* and *eno* encoded 6-phosphofructokinase 1 and enolase, respectively, and were the most important genes contributing to the reduced amino acid levels in the leaves of *C. paliuru*s at the S3 stage. The interactions between the bZIP TFs TRINITY_DN82089_c0_g2, TRINITY_DN89043_c4_g4, TRINITY_DN82089_c0_g3, and TRINITY_DN96843_c0_g2 might play an important role in the regulation of the biosynthesis of amino acids in the leaves of *C. paliurus* during the developmental stages. These results may help to elucidate amino acid biosynthesis during the development of leaves of *C. paliurus*.

## Supplemental Information

10.7717/peerj.13689/supp-1 Supplemental Information 1 The components of identified metabolites in the leaves of *Cyclocarya paliurus* at the S1, S2 and S3 developmental stages.

10.7717/peerj.13689/supp-2 Supplemental Information 2 PCA results of the relative abundance of free amino acid contents in the leaves of *Cyclocarya paliurus* at different developmental stages. S1, S2, and S3 indicate different developmental stages. Mix indicates the quality control sample.

10.7717/peerj.13689/supp-3 Supplemental Information 3 Heat map of DEGs related to amino acid biosynthesis in the leaves of *Cyclocarya paliurus* over different developmental stages. The log_2_ transformed values of DEGs are indicated from blue to red (low to high) across the S1, S2 and S3 developmental stages.

10.7717/peerj.13689/supp-4 Supplemental Information 4 PCA results of DEGs in the leaves of *Cyclocarya paliurus* at different developmental stages.

10.7717/peerj.13689/supp-5 Supplemental Information 5 Real-time PCR validation of different candidate unigenes involved in *Cyclocarya paliurus* amino acids biosynthesis by RNA-seq. The histogram shows the relative gene expression obtained *via* real-time PCR. The transcripts per million (TPM) of each million mapped fragments of the transcriptome are represented by a line graph. The right *y*-axis indicates gene expression levels calculated as TPM. The left *y*-axis indicates relative gene expression levels obtained *via* real-time PCR.

10.7717/peerj.13689/supp-6 Supplemental Information 6 Correlation analysis of the gene expression ratios between RT-qPCR and RNA-seq. 2−^ΔΔT^method was employed to calculate the relative gene expression levels obtained *via* real time PCR. The x-axis indicates base 10 logarithmic values of the relative gene expression levels. The y-axis indicates gene expression levels calculated as lgTPM.

10.7717/peerj.13689/supp-7 Supplemental Information 7 The characteristics of the leaves collected at different developmental stage (S1, S2 and S3).

10.7717/peerj.13689/supp-8 Supplemental Information 8 RT-qPCR primers used in this study.

10.7717/peerj.13689/supp-9 Supplemental Information 9 Information and relative abundance of all metabolites.

10.7717/peerj.13689/supp-10 Supplemental Information 10 Information and relative abundance of amino acids.

10.7717/peerj.13689/supp-11 Supplemental Information 11 Annotation of all unigenes.

10.7717/peerj.13689/supp-12 Supplemental Information 12 DEGs involved in the amino acid biosynthesis pathway.

10.7717/peerj.13689/supp-13 Supplemental Information 13 Correlation between genes and metabolites (*p*-value ≤ 0.05, |PCC| ≥ 0.8).

10.7717/peerj.13689/supp-14 Supplemental Information 14 Raw data for RT-qPCR.

10.7717/peerj.13689/supp-15 Supplemental Information 15 Differentially expressed bZIP genes screened in PlantTFDB database.

## Additional Information and Declarations

### Data Availability

The following information was supplied regarding data availability: The sequences are available at the National Center for Biotechnology Information (NCBI): PRJNA548403. This Transcriptome Shotgun Assembly project is available at DDBJ/ENA/GenBank: GJSE00000000. The other data are available in the Supplemental Tables.
