# Supplementary figures and images for: Amino acids profiling and transcriptomic data integration demonstrates the dynamic regulation of amino acids synthesis in the leaves of Cyclocarya paliurus
Source: PeerJ. 2022 Jul 5;10:e13689. doi: 10.7717/peerj.13689 (PMC9266588; doi:10.7717/peerj.13689)

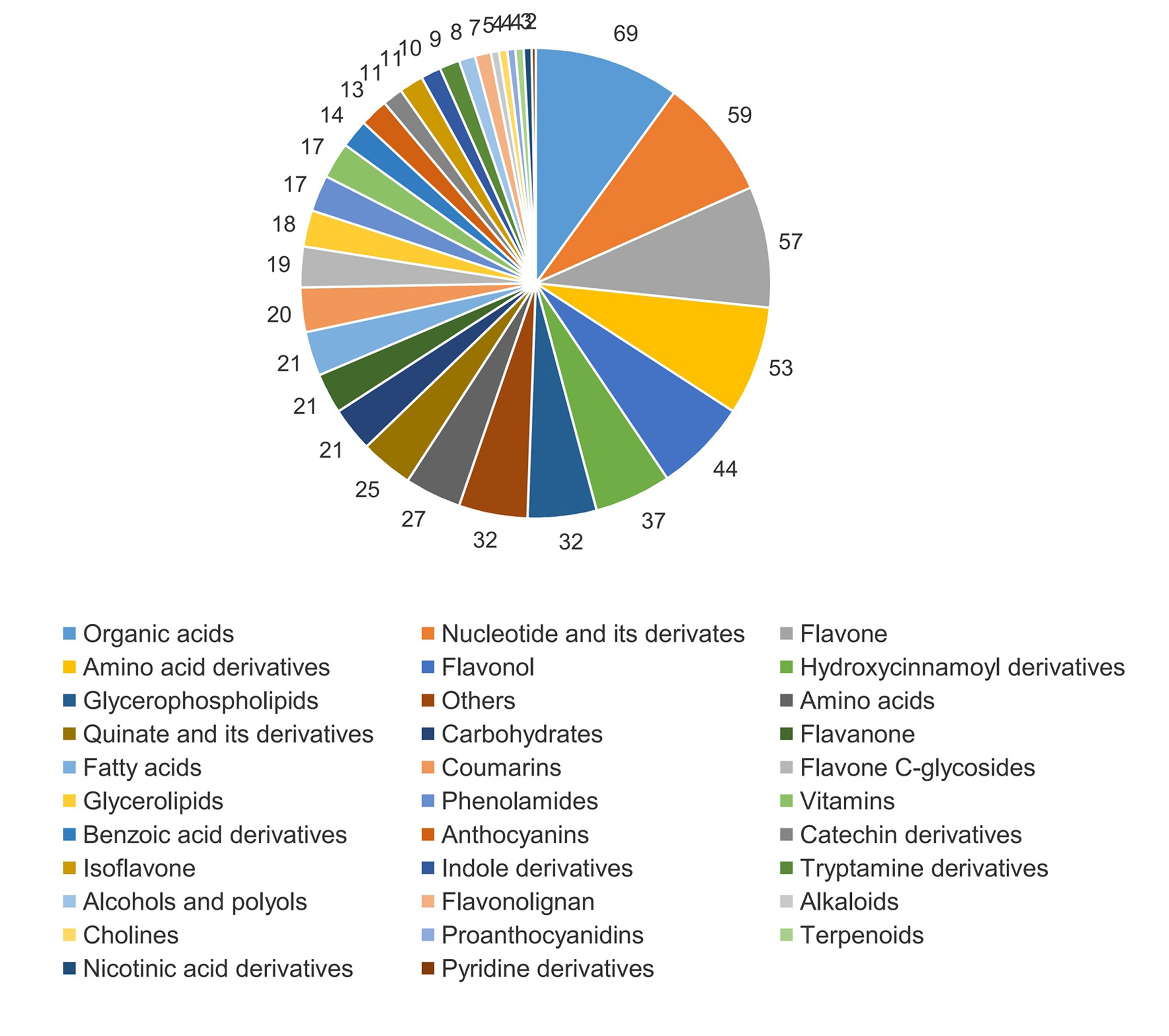

Supplement: Supplemental Information 1 [file peerj-10-13689-s001.jpg]

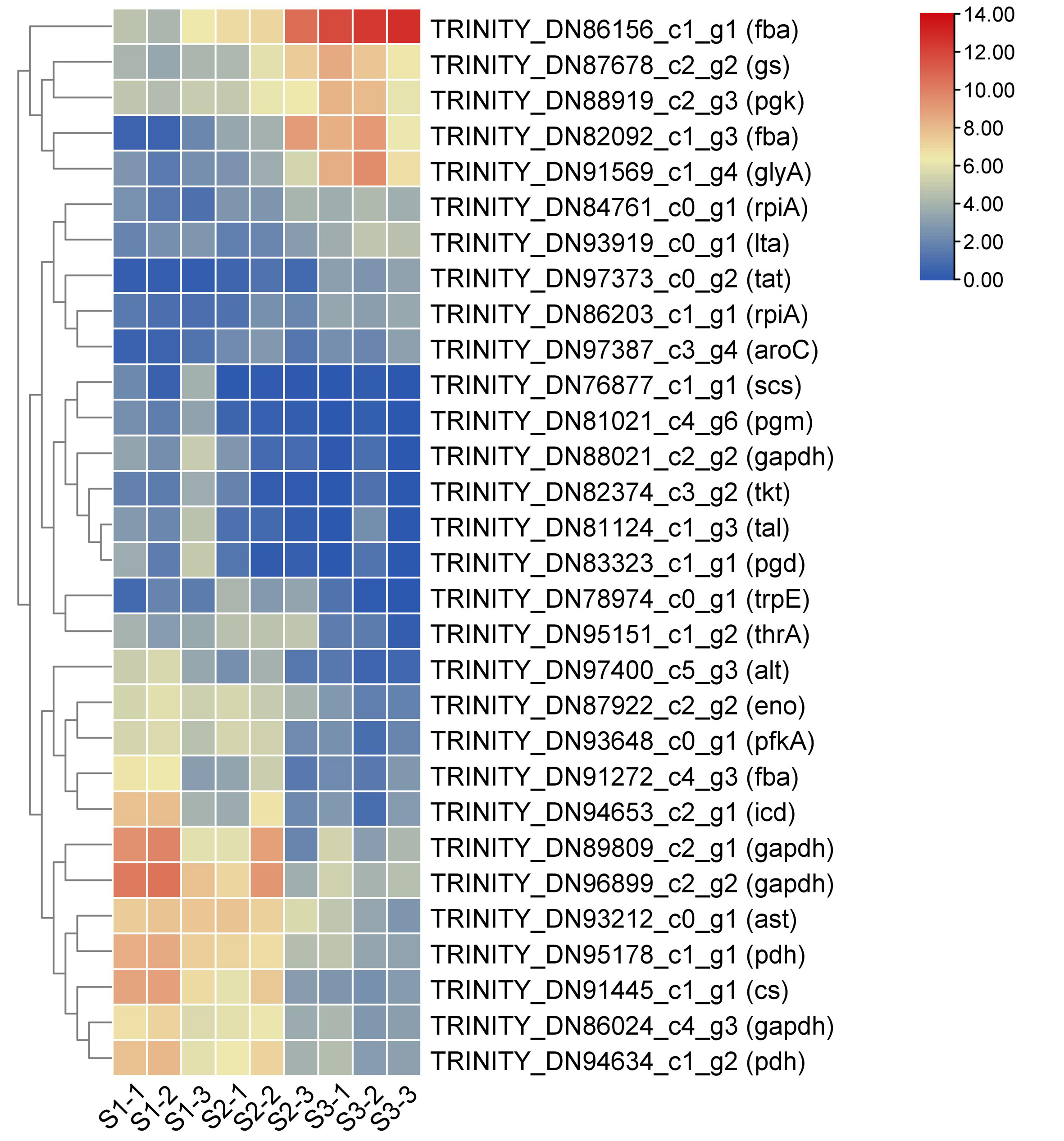

Supplement: Supplemental Information 2 — S1, S2, and S3 indicate different developmental stages. Mix indicates the quality control sample. [file peerj-10-13689-s002.jpg]

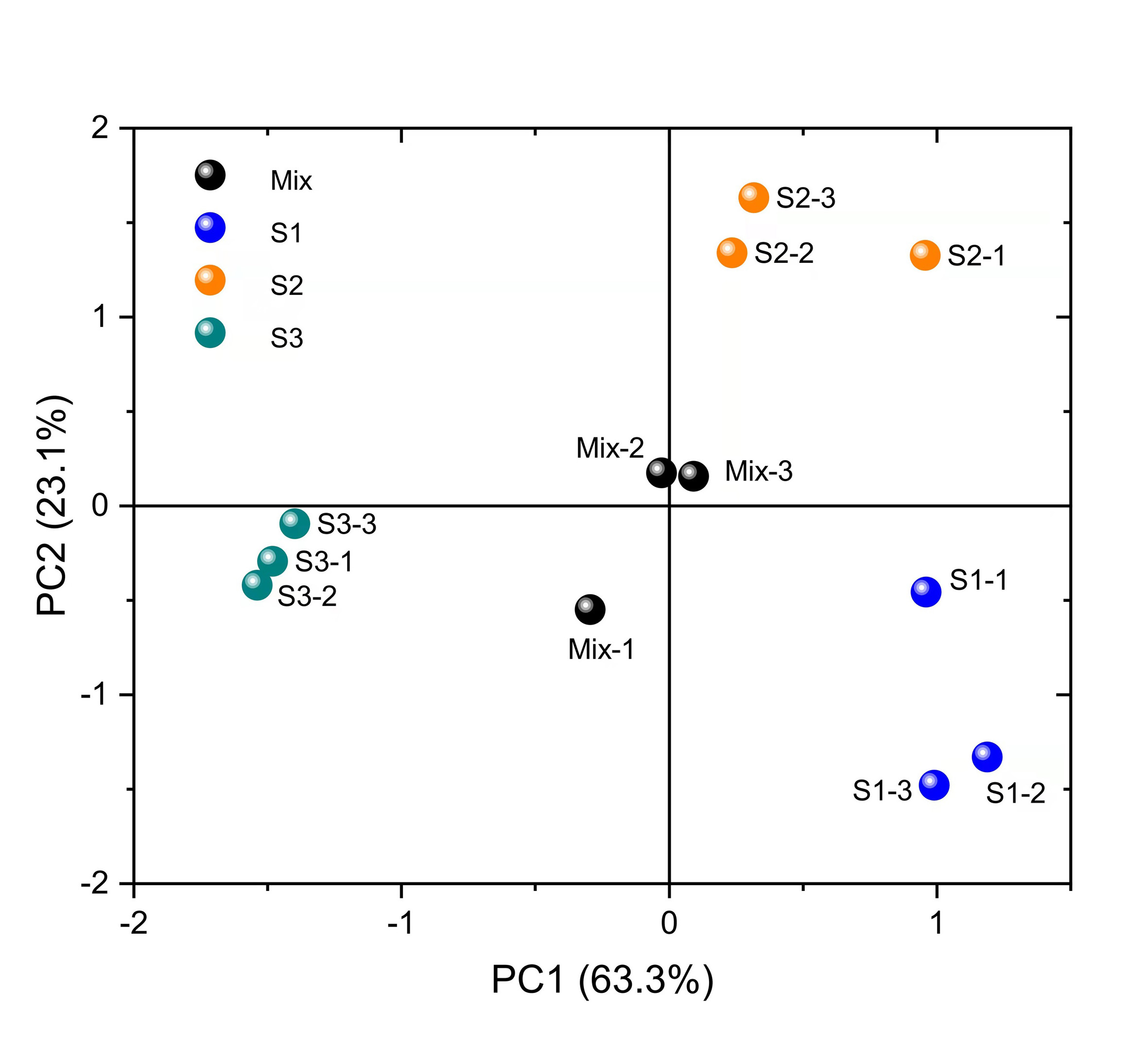

Supplement: Supplemental Information 3 — The log2 transformed values of DEGs are indicated from blue to red (low to high) across the S1, S2 and S3 developmental stages. [file peerj-10-13689-s003.jpg]

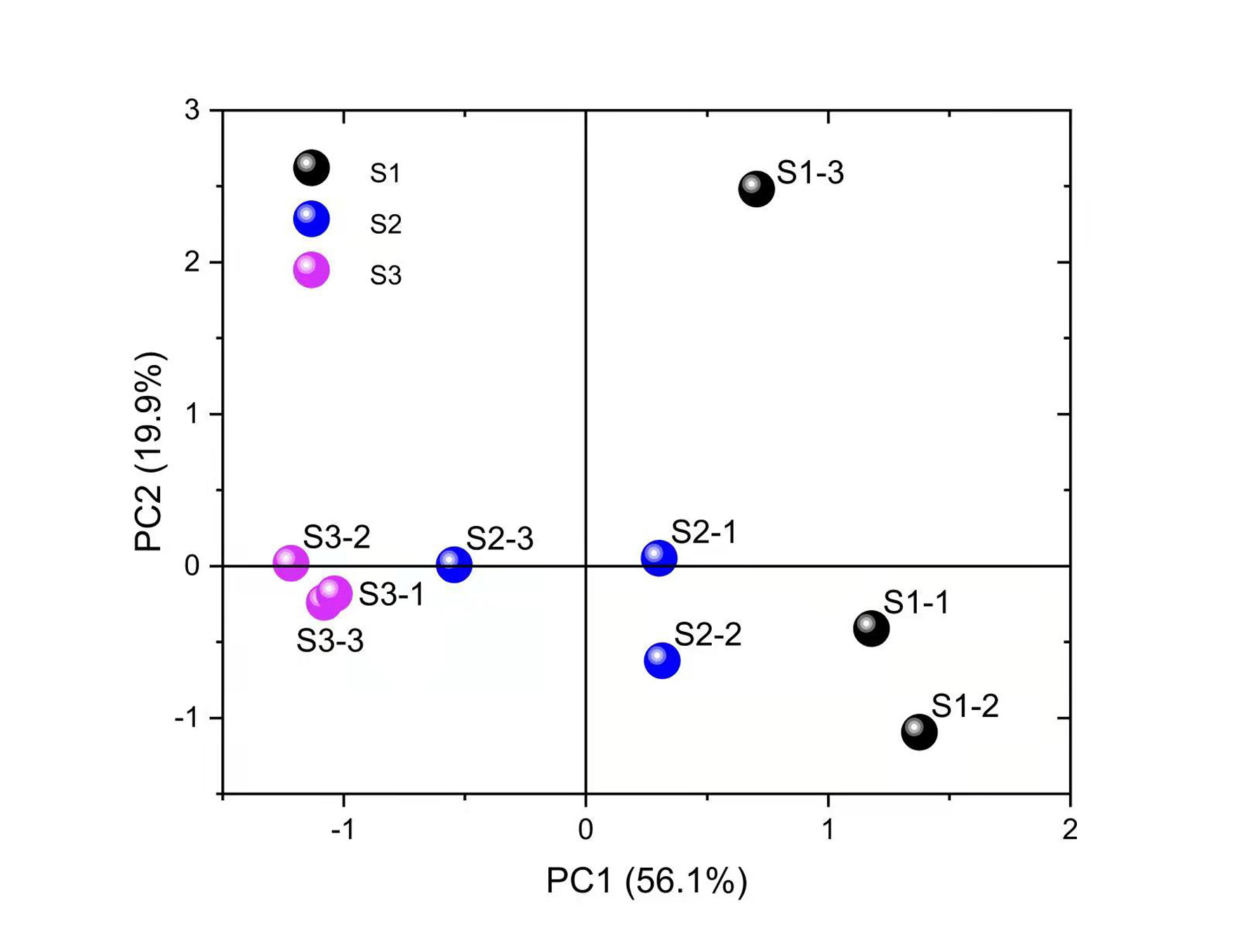

Supplement: Supplemental Information 4 [file peerj-10-13689-s004.jpg]

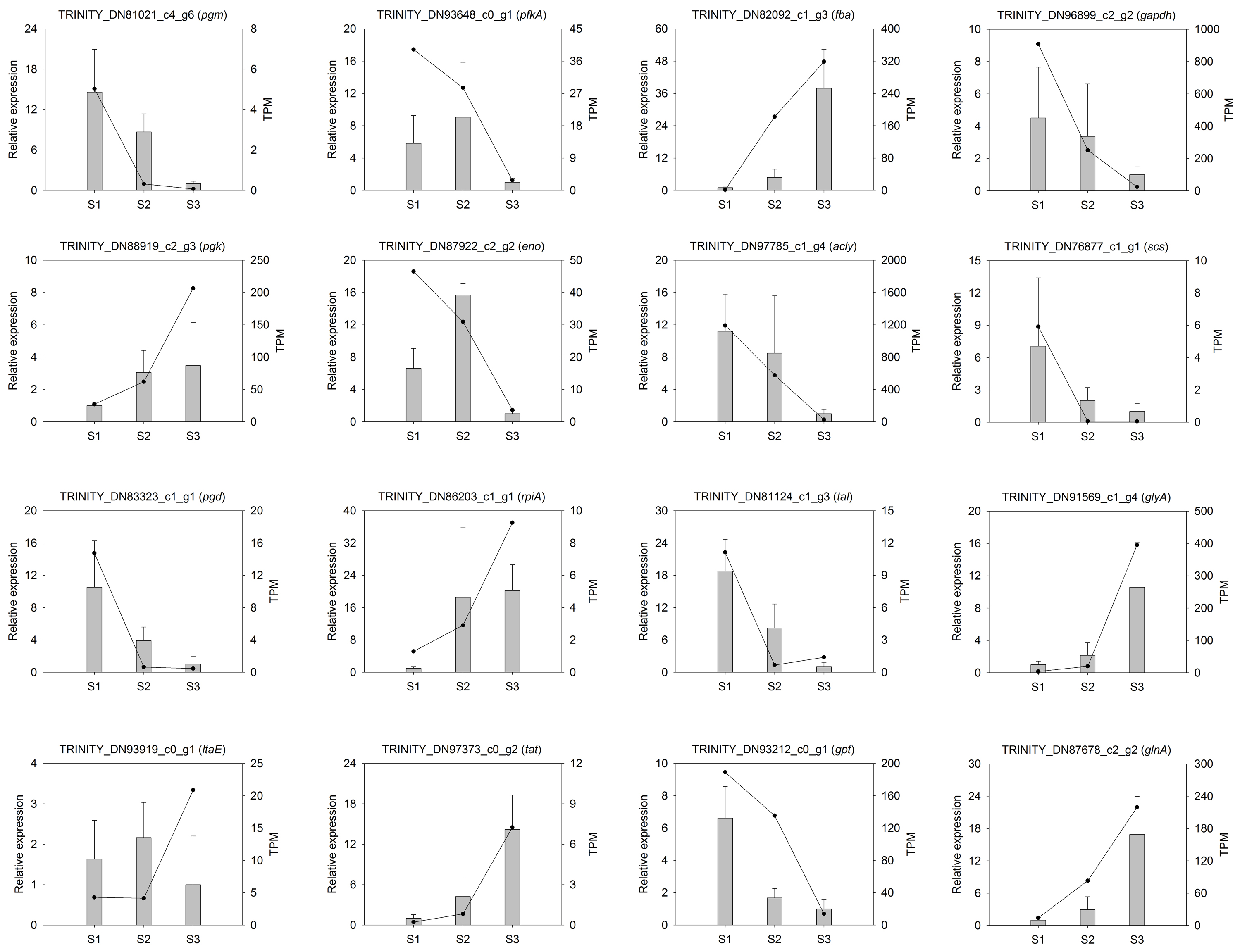

Supplement: Supplemental Information 5 — The histogram shows the relative gene expression obtained via real-time PCR. The transcripts per million (TPM) of each million mapped fragments of the transcriptome are represented by a line graph. The right y-axis indicates gene expression levels calculated as TPM. The left y-axis indicates relative gene expression levels obtained via real-time PCR. [file peerj-10-13689-s005.jpg]

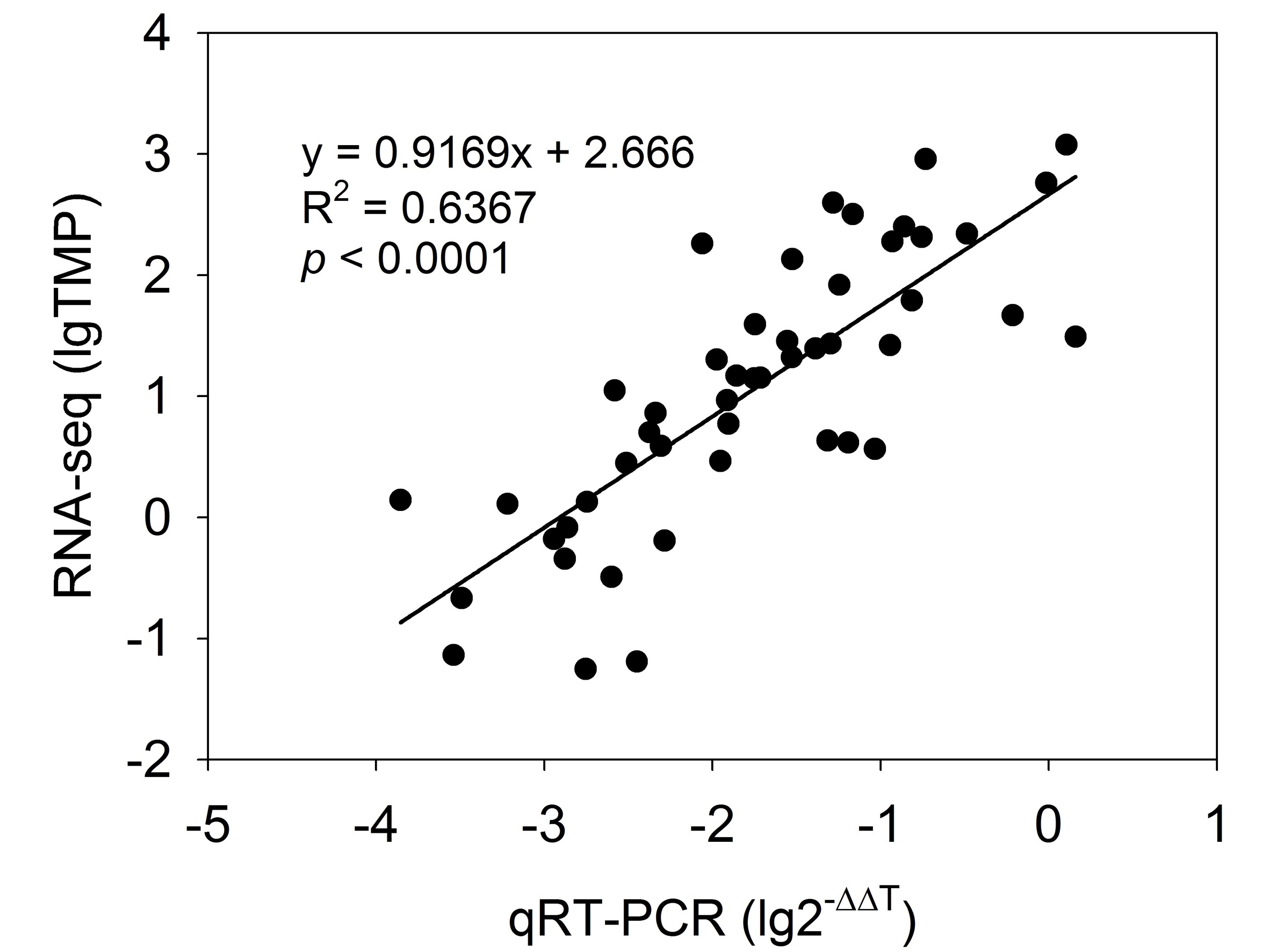

Supplement: Supplemental Information 6 — 2−ΔΔTmethod was employed to calculate the relative gene expression levels obtained via real time PCR. The x-axis indicates base 10 logarithmic values of the relative gene expression levels. The y-axis indicates gene expression levels calculated as lgTPM. [file peerj-10-13689-s006.jpg]
