# Supplementary material for: Amino acids profiling and transcriptomic data integration demonstrates the dynamic regulation of amino acids synthesis in the leaves of Cyclocarya paliurus
Source: PeerJ. 2022 Jul 5;10:e13689. doi: 10.7717/peerj.13689 (PMC9266588; doi:10.7717/peerj.13689)
Supplement: Supplemental Information 7 [file peerj-10-13689-s007.docx]

Table S1 The characteristics of the leaves collected at different developmental stage (S1, S2 and S3).

| Stage | Area (cm^2^) | Width (cm) | Length (cm) | Perimeter (cm) |
| --- | --- | --- | --- | --- |
| S1 | 1.45±0.65a | 1.79±0.64a | 1.28±0.56a | 5.39±0.86a |
| S2 | 3.44±1.08b | 1.95±1.00a | 2.63±0.56b | 8.06±1.03b |
| S3 | 35.28±4.79c | 4.72±0.31b | 10.29±1.00c | 27.07±2.11c |
